# Use of Medication for Opioid Use Disorder Among US Adolescents and Adults With Need for Opioid Treatment, 2019

**DOI:** 10.1001/jamanetworkopen.2022.3821

**Published:** 2022-03-23

**Authors:** Pia M. Mauro, Sarah Gutkind, Erin M. Annunziato, Hillary Samples

**Affiliations:** 1 Department of Epidemiology, Columbia University Mailman School of Public Health, New York, New York; 2 Center for Health Sciences Research, Rutgers Institute for Health, Health Care Policy and Aging Research, New Brunswick, New Jersey; 3 Department of Health Behavior, Society and Policy, Rutgers School of Public Health, Piscataway, New Jersey

## Abstract

**Importance:**

Medication for opioid use disorder (MOUD) is the criterion standard treatment for opioid use disorder (OUD), but nationally representative studies of MOUD use in the US are lacking.

**Objective:**

To estimate MOUD use rates and identify associations between MOUD and individual characteristics among people who may have needed treatment for OUD.

**Design, Setting, and Participants:**

Cross-sectional, nationally representative study using the 2019 National Survey on Drug Use and Health in the US. Participants included community-based, noninstitutionalized adolescent and adult respondents identified as individuals who may benefit from MOUD, defined as (1) meeting criteria for a past-year OUD, (2) reporting past-year MOUD use, or (3) receiving past-year specialty treatment for opioid use in the last or current treatment episode.

**Main Outcomes and Measures:**

The main outcomes were treatment with MOUD compared with non-MOUD services and no treatment. Associations with sociodemographic characteristics (eg, age, race and ethnicity, sex, income, and urbanicity); substance use disorders; and past-year health care or criminal legal system contacts were analyzed. Multinomial logistic regression was used to compare characteristics of people receiving MOUD with those receiving non-MOUD services or no treatment. Models accounted for predisposing, enabling, and need characteristics.

**Results:**

In the weighted sample of 2 206 169 people who may have needed OUD treatment (55.5% male; 8.0% Hispanic; 9.9% non-Hispanic Black; 74.6% non-Hispanic White; and 7.5% categorized as non-Hispanic other, with other including 2.7% Asian, 0.9% Native American or Alaska Native, 0.2% Native Hawaiian or Pacific Islander, and 3.8% multiracial), 55.1% were aged 35 years or older, 53.7% were publicly insured, 52.2% lived in a large metropolitan area, 56.8% had past-year prescription OUD, and 80.0% had 1 or more co-occurring substance use disorders (percentages are weighted). Only 27.8% of people needing OUD treatment received MOUD in the past year. Notably, no adolescents (aged 12-17 years) and only 13.2% of adults 50 years and older reported past-year MOUD use. Among adults, the likelihood of past-year MOUD receipt vs no treatment was lower for people aged 50 years and older vs 18 to 25 years (adjusted relative risk ratio [aRRR], 0.14; 95% CI, 0.05-0.41) or with middle or higher income (eg, $50 000-$74 999 vs $0-$19 999; aRRR, 0.18; 95% CI, 0.07-0.44). Compared with receiving non-MOUD services, receipt of MOUD was more likely among adults with at least some college (vs high school or less; aRRR, 2.94; 95% CI, 1.33-6.51) and less likely in small metropolitan areas (vs large metropolitan areas, aRRR, 0.41; 95% CI, 0.19-0.93). While contacts with the health care system (85.0%) and criminal legal system (60.5%) were common, most people encountering these systems did not report receiving MOUD (29.5% and 39.1%, respectively).

**Conclusions and Relevance:**

In this cross-sectional study, MOUD uptake was low among people who could have benefited from treatment, especially adolescents and older adults. The high prevalence of health care and criminal legal system contacts suggests that there are critical gaps in care delivery or linkage and that cross-system integrated interventions are warranted.

## Key Points

**Question:**

What are the individual characteristics associated with medication for opioid use disorder (MOUD) receipt among people with opioid use disorder treatment need?

**Findings:**

In this cross-sectional study with a weighted sample of 2 206 169 people with treatment need, approximately 1 in 4 (27.8%) reported past-year MOUD use, including no adolescents and only 13.2% of adults 50 years and older. Use of MOUD was low despite high prevalence of past-year health care or criminal legal system contacts.

**Meaning:**

Given that MOUD use was low, these results suggest that cross-system integrated interventions to increase MOUD uptake are needed, especially for younger age groups and older adults.

## Teaser

This cross-sectional study of respondents to the National Survey on Drug Use and Health evaluates rates of medication for opioid use disorder receipt among people with need for treatment as well as associated sociodemographic characteristics.

## Introduction

In 2019, 70.6% of the 70 630 drug overdose fatalities in the US involved opioids.^1^ These premature deaths have been associated with millions of years of life lost, including more than 1.6 million life-years attributed to opioid-related deaths in 2016 alone.^2^ Opioid-related deaths can be prevented through overdose reversal medication (ie, naloxone)^3^ and upstream treatment of underlying opioid use disorder (OUD).^4^ Evidence supporting the effectiveness of medication for OUD (MOUD; ie, methadone, buprenorphine, or naltrexone)^5,6,7^ is unequivocal, making it the criterion standard OUD treatment. Medication for OUD is associated with reductions in opioid use^8^ and disorder,^9^ longer treatment retention,^10,11^ and substantially reduced opioid-related mortality.^7^ Despite the strong evidence base, access to MOUD is limited by low facility and clinician uptake^12,13,14,15^ and persistent stigma surrounding OUD and medication.^16,17,18^

Current estimates of MOUD use rely on administrative data, such as specialty substance use treatment episodes^10,19,20^ and insurance or prescription records.^12,21,22,23,24,25^ These estimates consistently indicate low access to MOUD, particularly among younger age groups,^7,12,20,21^ pregnant women,^22^ residents of rural counties,^26^ adults involved in the criminal legal system,^1,27,28^ and racial and ethnic minority individuals.^23^ Studies have described individual and contextual characteristics associated with OUD treatment broadly^29,30^ but have not examined MOUD specifically owing to a lack of nationally representative data. As a result, knowledge about MOUD use is limited to convenience or clinical samples, which may differ systematically from people with OUD treatment need in the general population.

In 2019, the National Survey on Drug Use and Health (NSDUH) began measuring MOUD use, which for the first time made it possible to obtain nationally representative estimates of MOUD using a community-based sample.^31^ However, little is known about individual-level characteristics associated with past-year MOUD. This gap is even wider for individuals without medically documented OUD diagnoses because they are often excluded from research using clinical and administrative samples. Examining MOUD among a more expansive sample of people who may need treatment is clinically meaningful because people receiving MOUD may no longer meet OUD criteria or, alternatively, may receive medication without a diagnosis.^24,32^

This study is the first to our knowledge to estimate past-year MOUD use in a nationally representative community sample of people who may have needed past-year OUD treatment, which included noninstitutionalized people with OUD or who reported treatment for opioids. Building on past studies examining OUD treatment services more generally,^29,30^ we compared characteristics of people receiving MOUD with those of people receiving non-MOUD services (ie, connected with treatment but not receiving medication) or no treatment at all. We hypothesized that MOUD use would be particularly low among younger age groups^20,21^ and would be disproportionately distributed by sex, race and ethnicity, and urbanicity, based on previous research.^25,29^ We also identified points of treatment engagement, describing MOUD among people in contact with the health care and criminal legal systems, to inform interventions aimed at improving treatment access. To our knowledge, this is the first study to quantify MOUD use for the general population with OUD treatment need, providing critical evidence to build a more comprehensive understanding of care access and quality. Findings can inform national efforts needed to increase equitable access to MOUD in the US.

## Methods

### Data Source

The NSDUH is an annual, nationally representative cross-sectional household survey of people 12 years and older in the US designed to provide estimates of substance use and disorders. The complex survey design captured households in all 50 states, excluding people who were institutionalized or homeless and not in shelters.^33^ In-person interviews incorporated audio computer-assisted self-interviewing to increase willingness to report sensitive behaviors honestly.^33^ Drug use disorder measures had moderate validity and reliability (κ = 0.60-0.67).^34,35^ The NSDUH was approved by the RTI institutional review board.^36^ In 2019, the total response rate was 45.8%, including 70.5% for screening and 64.9% for weighted interviews.^33,36^ The Columbia University institutional review board approved this study; the use of deidentified public-use data was not considered human participants research and did not require informed consent beyond what was provided through the NSDUH. This study followed the Strengthening the Reporting of Observational Studies in Epidemiology (STROBE) reporting guidelines for cross-sectional studies.^37^

### Study Sample

The 2019 NSDUH deidentified public-use data included 56 136 people 12 years and older in the US. Inclusion criteria for past-year OUD treatment need were as follows: (1) past-year OUD (ie, past-year *Diagnostic and Statistical Manual of Mental Disorders* [Fourth Edition]^38^ heroin or prescription pain reliever abuse or dependence); (2) past-year MOUD (ie, “medication to help reduce or stop your use of [heroin/prescription pain relievers]”), or (3) past-year or current specialty treatment episode for heroin or prescription pain relievers (eMethods in the Supplement, measures 1-4, for wording and question sequence). These criteria adapted the NSDUH “treatment need” definition^33^ to be OUD-specific, including MOUD. Our final unweighted sample was n = 487. Of all observations not meeting the inclusion criteria, 1.9% were excluded owing to unknown or missing inclusion measure responses (ie, “don’t know,” refused, blank, or “bad data”; eMethods in the Supplement).

### Measures

#### Past-Year MOUD, Non-MOUD Services, or No Treatment

Participants reporting lifetime use of heroin or nonmedical prescription opioids and past-year treatment for drug use were asked about past-year MOUD (eg, buprenorphine, methadone, or naltrexone; eMethods in the Supplement, measure 5). Past-year MOUD indicated using “medication to help reduce or stop your use of [heroin/prescription pain relievers].” Non-MOUD services included reporting past-year treatment for drug use in any setting among those not reporting MOUD. We created a 3-level categorical OUD treatment variable (eMethods in the Supplement, measure 5): past-year MOUD, non-MOUD services (ie, past-year treatment or counseling for drug use but no MOUD), and no treatment (ie, no past-year MOUD or non-MOUD services).

#### Individual Predisposing, Enabling, and Need Characteristics

We used the Andersen Behavioral Model of Health Services Use^39^ and previous literature^21^ to select individual characteristics of clinical interest, as categorized in the public-use NSDUH. *Predisposing characteristics* included age (12-17, 18-25, 26-34, 35-49, or ≥50 years), sex (male or female), and education (among adults: high school or less or at least some college). Self-reported race and ethnicity included Hispanic/Latinx, non-Hispanic Black, non-Hispanic White, and non-Hispanic other (including Asian, Native American or Alaska Native, Native Hawaiian or Pacific Islander, and/or multiracial). *Enabling resources* included household income (<$20 000, $20 000-$49 999, $50 000-$74 999, or ≥$75 000), insurance status (any public insurance [eg, Medicaid, the Children’s Health Insurance Program, Medicare, or the Civilian Health and Medical Program of the Uniformed Services or other military insurance], private only, or uninsured or other), and urbanicity (large, small, or nonmetropolitan area). *Need* variables included OUD (*Diagnostic and Statistical Manual of Mental Disorders* [Fourth Edition]^38^ heroin and prescription pain reliever abuse or dependence), any other co-occurring substance use disorder (excluding opioid or tobacco use disorder), and past-year major depressive episode.

#### Contacts With Health Care and Criminal Legal Systems

Health care contacts included past-year health care use in emergency, inpatient, or outpatient settings. Past-year criminal legal system contacts included any past-year booking, arrests, probation, or parole.

### Statistical Analysis

We calculated descriptive statistics for the past-year OUD treatment need sample using survey weights to derive nationally representative estimates, as well as the proportion within each subgroup reporting past-year MOUD, non-MOUD services, or no treatment. Then we fit multinomial regression models to identify the association of predisposing, enabling, and need variables with MOUD, as compared with both non-MOUD services and no drug treatment. We also examined past-year contacts with the health care and criminal legal systems to describe rates of MOUD among people encountering each system. In sensitivity analyses, the model included detailed categories of public insurance and an additional indicator of past-year criminal legal system involvement to examine associations independent of criminal legal contact. All analyses were conducted using svy command estimations in Stata, version 15MP (StataCorp LLC), with standard errors accounting for complex survey data design using Taylor linearization, and a 2-sided *P* value significance threshold of less than .05.

## Results

Among the weighted sample of 2 206 169 people (unweighted, 487) who may have needed OUD treatment (55.5% male; 8.0% Hispanic, 9.9% non-Hispanic Black, 74.6% non-Hispanic White, and 7.5% non-Hispanic other, with other including 2.7% Asian, 0.9% Native American or Alaska Native, 0.2% Native Hawaiian or Pacific Islander, and 3.8% identified as multiracial), 55.1% were aged 35 years or older, 53.7% were publicly insured, 52.2% lived in a large metropolitan area, 56.8% had past-year prescription OUD, and 80.0% had 1 or more co-occurring substance use disorders (percentages are weighted) (Table 1).

**Table 1.** Sociodemographic Characteristics of Adolescents and Adults With Past-Year OUD Treatment Need^a^

Characteristic	Overall sample of respondents with OUD treatment need		Past-year drug treatment use						*P* value
	Weighted No.	Weighted column % (95% CI)	No treatment		Non-MOUD services		MOUD		
			Weighted No.	Weighted row % (95% CI)	Weighted No.	Weighted row % (95% CI)	Weighted No.	Weighted row % (95% CI)	
Total (row %)	2 206 169	100.0	1 256 838	57.0 (50.9-62.8)	336 580	15.3 (12.0-19.2)	612 750	27.8 (22.0-34.3)	NA
Predisposing									
Age, y									
12-17^b^	92 081	4.2 (2.8-6.1)	80 829	87.8 (72.4-95.2)	11 252	12.2 (4.8-27.6)	0	0 (0-0)	<.001
18-25^b^	251 824	11.4 (8.8-14.7)	154 797	61.5 (46.4-74.6)	41 492	16.5 (10.4-25.1)	55 534	22.0 (12.3-36.3)	
26-34^b^	646 460	29.3 (23.6-35.8)	264 713	41.0 (28.6-54.6)	108 180	16.7 (9.1-28.7)	273 567	42.3 (30.7-54.8)	
35-49^b^	699 988	31.7 (26.6-37.4)	342 394	48.9 (40.8-57.1)	141 967	20.3 (13.5-29.3)	215 627	30.8 (22.6-40.5)	
≥50^b^	515 817	23.4 (16.3-32.4)	414 106	80.3 (64.8-90.0)	33 689	6.5 (3.8-11.0)	68 022	13.2 (5.8-27.4)	
Sex									
Male	1 224 910	55.5 (49.4-61.5)	658 927	53.8 (45.9-61.5)	231 657	18.9 (14.0-25.0)	334 326	27.3 (20.0-36.0)	.10
Female	981 259	44.5 (38.5-50.6)	597 911	60.9 (52.3-68.9)	104 923	10.7 (6.9-16.2)	278 425	28.4 (21.8-36.0)	
Race and ethnicity									
Hispanic	176 701	8.0 (5.8-11.0)	126 733	71.7 (44.2-89.0)	23 835	13.5 (6.5-25.8)	26 133^c^	14.8 (2.9-50.0)	.60
Non-Hispanic									
Black	219 095	9.9 (6.7-14.5)	138 214	63.1 (43.9-78.9)	38 178	17.4 (7.0-37.0)	42 703	19.5 (9.2-36.6)	
White	1 645 196	74.6 (68.5-79.8)	881 641	53.6 (46.3-60.8)	253 883	15.4 (11.2-20.8)	509 672	31.0 (23.9-39.1)	
Other	165 178	7.5 (4.4-12.4)	110 251	66.8 (41.6-85.0)	20 684^c^	12.5 (4.3-31.1)	34 243^c^	20.7 (7.2-47.0)	
Education (≥18 y)									
High school or less^b^	935 320	44.2 (37.5-51.2)	470 296	50.3 (42.5-58.1)	206 670	22.1 (15.6-30.3)	258 354	27.6 (20.0-36.8)	.03
Some college or more^b^	1 178 768	55.8 (48.8-62.5)	705 714	59.9 (50.8-68.5)	118 658	10.1 (06.0-16.5)	354 396	30.1 (23.2-37.9)	
Enabling									
Insurance									
Any public^b^	1 185 665	53.7 (46.9-60.4)	585 286	49.4 (40.9-57.9)	183 580	15.5 (11.4-20.8)	416 799	35.2 (26.6-44.8)	.01
Private only^b^	576 544	26.1 (19.7-33.8)	393 950	68.3 (57.7-77.3)	61 249	10.6 (6.1-17.8)	121 345	21.0 (12.3-33.6)	
Uninsured/other^b^	443 960	20.1 (14.6-27.1)	277 603	62.5 (49.5-74.0)	91 751	20.7 (12.8-31.7)	74 606	16.8 (9.2-28.8)	
Income, $									
0-19 999	736 805	33.4 (26.3-41.4)	321 047	43.6 (32.9-54.9)	138 994	18.9 (11.7-28.9)	276 765	37.6 (28.1-48.0)	.09
20 000-49 999	674 355	30.6 (24.0-38.0)	407 270	60.4 (48.6-71.1)	99 968	14.8 (8.6-24.3)	167 117	24.8 (15.6-37.1)	
50 000-74 999	311 849	14.1 (10.3-19.0)	209 007	67.0 (52.3-79.0)	52 298	16.8 (8.2-31.2)	50 544	16.2 (9.8-25.6)	
≥75 000	483 160	21.9 (15.5-30.0)	319 514	66.1 (50.8-78.7)	45 321	9.4 (3.9-20.9)	118 325	24.5 (13.5-40.2)	
Urbanicity									
Large metropolitan	1 152 707	52.2 (46.3-58.1)	690 354	59.9 (52.4-67.0)	127 075	11.0 (6.4-18.5)	335 278	29.1 (22.2-37.1)	.30
Small metropolitan	754 477	34.2 (28.9-40.0)	394 378	52.3 (40.5-63.8)	165 656	22.0 (15.4-30.3)	194 443	25.8 (18.0-35.5)	
Nonmetropolitan	298 985	13.6 (9.8-18.4)	172 106	57.6 (38.1-74.9)	43 849	14.7 (7.5-26.6)	83 029	27.8 (14.6-46.4)	
Need									
Any OUD^b^	1 700 870	77.1 (71.3-82.0)	1 256 838	73.9 (67.9-79.1)	150 072	8.8 (6.4-12.0)	293 959	17.3 (12.6-23.3)	<.001
Prescription OUD only^b^	1 253 326	56.8 (51.0-62.5)	1 049 077	83.7 (75.7-89.4)	77 281	6.2 (3.2-11.5)	126 968	10.1 (6.0-16.6)	<.001
Heroin use disorder only^b^	267 312	12.1 (8.9-16.3)	157 826	59.0 (44.1-72.5)	42 938	16.1 (7.2-32.2)	66 548	24.9 (14.5-39.3)	
Co-occurring heroin/prescription OUD^b^	180 232	8.2 (5.4-12.2)	49 936	27.7 (13.8-47.9)	29 853^c^	16.6 (6.0-38.3)	100 443	55.7 (35.2-74.5)	
Other co-occurring substance use disorder^b^	1 764 273	80.0 (73.4-85.3)	1 183 527	67.1 (60.8-72.8)	219 392	12.4 (9.2-16.5)	361 354	20.5 (14.8-27.6)	<.001
Major depressive episode (≥18 y)	852 697	38.6 (32.0-45.8)	439 950	51.6 (41.8-61.3)	129 214	15.2 (9.5-23.3)	283 534	33.2 (24.4-43.4)	.27

Abbreviations: MOUD, medication for opioid use disorder; NA, not applicable; OUD, opioid use disorder.

^a^ Weighted No. is the survey-weighted sample size; unweighted N = 487; weighted column % indicates survey-weighted column percentage; weighted percentages may not sum to 100 because of rounding. A callout of 18 years and older indicates the characteristic being restricted to adults 18 years and older. Other co-occurring substance use disorder includes 1 or more of the following past-year substance use disorders: alcohol, cannabis, cocaine, hallucinogens, inhalants, methamphetamine, tranquilizers, stimulants, sedatives, and psychedelics. Self-reported race and ethnicity included Hispanic/Latinx, non-Hispanic Black, non-Hispanic White, and non-Hispanic other (eg, Asian, Native American or Alaska Native, Native Hawaiian, Pacific Islander, or multiracial).

^b^ Design-based *P* less than .05 with Rao-Scott adjustment.

^c^ Lower bound of confidence interval for weighted sample estimate includes zero.

Only 27.8% of people needing OUD treatment received MOUD in the past year; 57.0% received no treatment, and 15.3% received non-MOUD services (Table 1). Notably, no adolescents (aged 12-17 years) and only 13.2% of adults 50 years and older reported past-year MOUD use. A minority of adults with higher education (30.1%) and high school or less education (27.6%) reported receiving MOUD. Other predisposing characteristics were not statistically associated with treatment status but signaled potential treatment disparities. For example, 14.8% of Hispanic respondents, 19.5% of non-Hispanic Black respondents, and 20.7% of respondents identified as other race and ethnicity reported receiving MOUD, compared with 31.0% of non-Hispanic White people. Insurance was the main enabling resource associated with treatment status. More than one-third of people with public insurance (35.2%) reported receiving MOUD compared with 21.0% with private coverage and 16.8% with no public or private insurance. Need variables were associated with MOUD use, including co-occurring substance use disorders. Overall, 17.3% of people with any OUD reported receiving MOUD, but a gradient was observed by OUD type, with only 10.1% for prescription OUD only, 24.9% for heroin use disorder only, and 55.7% for both heroin and prescription OUD reporting MOUD (percentages are weighted) (Table 1). Among people reporting MOUD, 52.0% did not meet past-year OUD criteria (eTable 1 in the Supplement).

Table 2 shows unadjusted and adjusted multinomial estimates comparing predisposing, enabling, and need characteristics of people receiving MOUD with people receiving no treatment or non-MOUD services. Adolescents were excluded because none reported past-year MOUD (eTable 2 in the Supplement, adult subsample characteristics). In adjusted models, groups less likely to report MOUD included people 50 years and older compared with ages 18 to 25 years (adjusted relative risk ratio [aRRR], 0.14; 95% CI, 0.05-0.41), people identifying as non-Hispanic other compared with people identifying as non-Hispanic White (aRRR, 0.28; 95% CI, 0.08-0.92), women compared with men (aRRR, 0.52; 95% CI, 0.29-0.95), people with private insurance only (aRRR, 0.34; 95% CI, 0.13-0.89) or no/other insurance (aRRR, 0.26; 95% CI, 0.08-0.87) compared with public insurance, and people reporting incomes $50 000 to $74 999 compared with less than $20 000 (aRRR, 0.18; 95% CI, 0.07-0.44). People with some college were more likely to report MOUD than those with high school or less education (aRRR, 2.12; 95% CI, 1.18-3.78). Need characteristics were strongly associated with MOUD, including significantly greater likelihood of MOUD for people with co-occurring heroin and prescription OUD (aRRR, 5.07; 95% CI, 1.50-17.12) and lower likelihood for other co-occurring substance use disorders (aRRR, 0.07; 95% CI, 0.03-0.16). Only 2 characteristics distinguished people receiving MOUD from those receiving non-MOUD services; MOUD was more likely among those with some college compared with lower education (aRRR, 2.94; 95% CI, 1.33-6.51) and less likely for people living in small compared with large metropolitan areas (aRRR, 0.41; 95% CI, 0.19-0.93).

**Table 2.** Likelihood of Medication for Opioid Use Disorder Among Adults Who May Have Needed OUD Treatment^a^

Characteristics	MOUD vs no treatment		MOUD vs non-MOUD services	
	uRRR (95% CI)	aRRR (95% CI)	uRRR (95% CI)	aRRR (95% CI)
Predisposing				
Age categories, y				
18-25	1 [Reference]	1 [Reference]	1 [Reference]	1 [Reference]
26-34	2.88 (1.11-7.50)^b^	1.37 (0.42-4.43)	1.89 (0.67-5.32)	1.30 (0.42-4.03)
35-49	1.76 (0.74-4.15)	0.84 (0.30-2.35)	1.13 (0.47-2.71)	0.75 (0.28-2.05)
≥50	0.46 (0.16-1.32)	0.14 (0.05-0.41)^b^	1.51 (0.60-3.79)	0.86 (0.31-2.42)
Race and ethnicity				
Hispanic	0.38 (0.06-2.39)	0.57 (0.14-2.28)	0.53 (0.09-3.00)	0.61 (0.15-2.57)
Non-Hispanic				
Black	0.60 (0.21-1.74)	0.82 (0.27-2.46)	0.57 (0.14-2.31)	0.52 (0.12-2.18)
White	1 [Reference]	1 [Reference]	1 [Reference]	1 [Reference]
Other	0.53 (0.13-2.16)	0.28 (0.08-0.92)^b^	0.80 (0.18-3.57)	0.35 (0.08-1.54)
Sex				
Male	1 [Reference]	1 [Reference]	1 [Reference]	1 [Reference]
Female	0.95 (0.57-1.56)	0.52 (0.29-0.95)^b^	1.87 (0.95-3.69)	1.77 (0.81-3.85)
Education				
High school or less	1 [Reference]	1 [Reference]	1 [Reference]	1 [Reference]
Some college or more	0.91 (0.57-1.46)	2.12 (1.18-3.78)^b^	2.39 (1.04-5.46)^b^	2.94 (1.33-6.51)^b^
Enabling				
Insurance				
Any public	1 [Reference]	1 [Reference]	1 [Reference]	1 [Reference]
Private only	0.45 (0.20-1.04)	0.34 (0.13-0.89)^b^	0.87 (0.30-2.51)	0.89 (0.23-3.40)
Uninsured/other	0.38 (0.18-0.81)^b^	0.26 (0.08-0.87)^b^	0.34 (0.15-0.79)^b^	0.33 (0.11-1.03)
Income, $				
0-19 999	1 [Reference]	1 [Reference]	1 [Reference]	1 [Reference]
20 000-49 999	0.49 (0.24-1.02)	0.47 (0.22-1.01)	0.86 (0.31-2.35)	0.72 (0.23-2.22)
50 000-74 999	0.28 (0.12-0.65)^b^	0.18 (0.07-0.44)^b^	0.47 (0.15-1.43)	0.35 (0.09-1.42)
≥75 000	0.44 (0.18-1.08)	0.37 (0.13-1.04)	1.27 (0.35-4.53)	0.83 (0.19-3.68)
Urbanicity				
Large metropolitan	1 [Reference]	1 [Reference]	1 [Reference]	1 [Reference]
Small metropolitan	0.98 (0.54-1.80)	0.95 (0.50-1.79)	0.45 (0.20-1.03)	0.41 (0.19-0.93)^b^
Nonmetropolitan	0.97 (0.37-2.54)	0.94 (0.32-2.78)	0.75 (0.25-2.23)	0.82 (0.26-2.58)
Need				
Co-occurring heroin and prescription OUD	4.42 (1.61-12.17)^b^	5.07 (1.50-17.12)^b^	1.94 (0.53-7.08)	2.31 (0.44-12.18)
Other co-occurring substance use disorder	0.10 (0.04-0.22)^b^	0.07 (0.03-0.16)^b^	0.81 (0.31-2.11)	0.64 (0.19-2.13)
Major depressive episode	1.44 (0.79-2.64)	1.58 (0.84-2.95)	1.31 (0.64-2.69)	0.98 (0.45-2.16)

Abbreviations: aRRR, adjusted relative risk ratio (from the multinomial model with a categorical outcome); MOUD, medication for opioid use disorder; OUD, opioid use disorder; uRRR, unadjusted relative risk ratio.

^a^ Weighted N = 2 114 089; unweighted N = 438. Adolescents aged 12 to 17 years were excluded from the model because of collinearity with the outcome. Other co-occurring substance use disorder includes 1 or more of the following past-year substance use disorders: alcohol, cannabis, cocaine, hallucinogens, inhalants, methamphetamine, tranquilizers, stimulants, sedatives, and psychedelics. Self-reported race/ethnicity included Hispanic/Latinx, non-Hispanic Black, non-Hispanic White, and non-Hispanic other (eg, Asian, Native American or Alaska Native, Native Hawaiian, Pacific Islander, or multiracial).

^b^ Design-based *P* < .05.

The Figure shows that 85.0% of the sample had past-year health care (ie, 80.8% outpatient, 20.6% inpatient, and 51.7% emergency department settings) or criminal legal system contacts (60.5%). Only a minority of people encountering these systems reported receiving MOUD (health, 29.5%; legal, 39.1%) (percentages are weighted).

**Figure.**
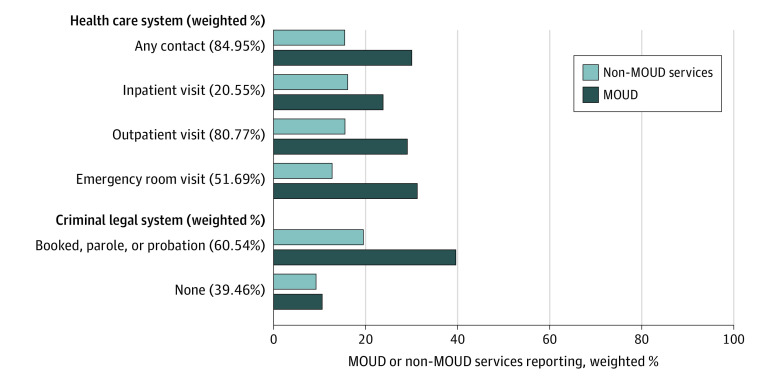
Past-Year Contact With Health Care and Criminal Legal System Contacts and Medication for Opioid Use Disorder (MOUD) Treatment, 2019 Weighted N = 2 206 169; unweighted N = 487.

Results of sensitivity analyses resembled the main results; criminal legal exposure was strongly associated with receiving MOUD compared with no treatment (aRRR, 3.73; 95% CI, 1.78-7.80; eTable 3 in the Supplement).

## Discussion

Our nationally representative cross-sectional study examined MOUD in a community sample of US adolescents and adults in 2019, extending prior studies using administrative data^12,40^ or examining OUD treatment broadly.^29,30^ Approximately 1 in 4 people who may have needed OUD treatment reported past-year MOUD. No adolescents received MOUD, and most adults received no drug treatment at all, indicating substantial gaps in access. While past-year OUD signaled clinical need for treatment, only 1 in 6 (17.3%) people with OUD reported receiving MOUD, although this was higher among people with co-occurring heroin and prescription OUD. Beyond clinical need, both predisposing and enabling characteristics were associated with MOUD compared with no treatment, but only education and urbanicity distinguished people receiving MOUD from those receiving non-MOUD services. This is consistent with prior work demonstrating that individual characteristics influence OUD treatment access^29^ and underscores the importance of key modifiable factors in distinguishing the type of treatment received.

Only adults reported receiving MOUD, consistent with past reports of low MOUD use in adolescents.^20,21,41^ Buprenorphine is approved by the US Food and Drug Administration for people 16 years and older and is the only medication approved to treat OUD in adolescents.^42^ Hesitancy about off-label prescribing for ages 12 to 15 years could contribute to underuse of MOUD in this population. Gaps in access could be worsened by specialty facilities with adolescent treatment programs being less likely to provide MOUD than facilities serving adults.^43^ Our findings support calls for additional MOUD engagement and retention strategies tailored for youths.^44,45^ Furthermore, older adults were less likely than young adults to receive MOUD, with most receiving no treatment at all. Misconceptions about substance use in older age alongside lower screening/assessment rates^46,47^ may contribute to the observed low OUD treatment rates.

Medication for OUD was lower among women after accounting for need and enabling characteristics. While this contrasts with previous literature that did not find differences by sex in OUD treatment use patterns more broadly,^29^ our findings were consistent with past work focused on MOUD.^25^ Our ability to detect statistical differences by race and ethnicity was limited because most people in the sample identified as non-Hispanic White, but MOUD use appeared racially patterned. Nearly one-third of non-Hispanic White people with OUD treatment need received MOUD, compared with approximately 20% of people identifying as non-Hispanic Black or other non-Hispanic or multiracial groups and 15% of Hispanic people. In contrast, roughly similar proportions of each racial and ethnic group received non-MOUD services, revealing substantial gaps specifically for MOUD access among people of color. In light of evidence showing faster growth in overdose death rates for minoritized groups^48^ and disparities in MOUD by community-level racial and ethnic composition,^49^ structural interventions that increase equitable MOUD access and retention are needed.^50^

Public insurance was an important enabling characteristic associated with MOUD, and sensitivity analyses showed that this association was driven by Medicaid. Therefore, policies that increase Medicaid coverage could be a key population-level strategy to enable MOUD.^50^ While all 50 states have Medicaid coverage for buprenorphine, as of 2018, only 42 states had Medicaid coverage for methadone.^51^ Starting in 2020, the Substance Use-Disorder Prevention that Promotes Opioid Recovery and Treatment (SUPPORT) for Patients and Communities Act mandated that Medicaid cover all 3 US Food and Drug Administration–approved medications for OUD, including methadone in certified opioid treatment programs.^52^ This important change could further increase MOUD in the publicly insured population because removing structural barriers, such as prior authorization policies,^53^ are important steps to increase MOUD use. The strong association of Medicaid coverage with MOUD could explain, in part, associations between lower income and MOUD, considering Medicaid is the primary source of insurance for low-income individuals in the US. These findings add to evidence highlighting the important role of public insurance in facilitating access to care for marginalized groups. However, our study shows that substantial gaps remain even among publicly insured people, which composed more than half of our sample.

While geography is associated with unequal distribution of MOUD prescribers,^54,55^ we found no differences by urbanicity for MOUD compared with no treatment. However, living in a small metropolitan area was associated with lower likelihood of MOUD compared with non-MOUD services. Individuals who receive treatment may have greater access to MOUD in urban areas with higher concentrations of prescribers, despite indications of county-level increases in buprenorphine prescribers across all categories of rurality.^56^ Growth in the number of clinicians with US Drug Enforcement Administration waivers required to prescribe buprenorphine is an important step to improve MOUD access, but more work is needed to increase prescribing rates among qualified clinicians^57,58,59,60^ and align prescribing practices with clinical guidelines.^61^

We found that health care and criminal legal system contacts were common, yet most people encountering these systems reported receiving no MOUD, highlighting systemic gaps and continued missed opportunities to increase MOUD uptake. Engaging people in care and initiating MOUD are the first stages in the OUD continuum of care and necessary to achieve the reductions in mortality and adverse opioid-related outcomes associated with MOUD retention.^8,25^ More than 80% had at least 1 general health care encounter, yet only 30% reported receiving MOUD. In a 2020 study,^46^ discussions about drug use with health care clinicians were associated with drug treatment use and perceived treatment need, indicating that relatively low-threshold interventions could potentially increase treatment uptake, yet these discussions were rare. Our findings provide further evidence that investments are needed to increase MOUD prescribing and referrals in ambulatory settings. Similarly, more than half of our sample reported a past-year emergency department visit, yet fewer than one-third of them reported receiving MOUD, supporting growing efforts to overcome barriers in implementing hospital-based MOUD induction and warm handoffs to community health care professionals.^62,63,64^ Consistent with past research,^65^ we found that criminal legal contact was associated with MOUD above and beyond other need, enabling, or predisposing characteristics. This could reflect mandated treatment or the continued criminalization of people who use drugs without necessarily linking people to treatment services. While we could not distinguish treatment referrals, many reports indicate low MOUD access through criminal legal settings.^19,66^ Strategies that do not require criminal legal contact to access drug treatment generally and MOUD specifically are needed to reinforce equitable community-based treatment access.

### Limitations

This study has limitations. While the NSDUH used audio computer-assisted self-interviewing to increase reporting of sensitive information and reduce social desirability bias, self-reported data may nonetheless underestimate drug use, particularly heroin.^67,68,69^ Our OUD treatment need inclusion definition was tailored to be OUD-specific, yet we may have missed people owing to data limitations,^70^ such as individuals with multiple past-year treatment episodes if the last episode treated a different substance. Because most people received no treatment, we expect this would be a small minority. Findings may not generalize to groups excluded from the survey, including institutionalized people in correctional settings who are disproportionately composed of racial and ethnic minoritized groups. Although we could not ascertain OUD treatment need and receipt for nonparticipating individuals, the NSDUH is the only available national data set measuring MOUD, making it an important source for national estimates. Findings should be interpreted alongside other OUD treatment need and MOUD indicators available. In addition, we could not differentiate the type of MOUD (eg, methadone vs buprenorphine) and call for future studies with restricted data access to examine differences by medication type.

## Conclusions

Despite strong evidence that medication is the most effective treatment for OUD and high rates of contact with the health care system, all adolescents and most adults with OUD treatment need in this study reported no past-year MOUD use. An important first step in understanding correlates of MOUD use in the general US population, this nationally representative study revealed critical gaps in treatment engagement and MOUD use, suggesting that increased efforts to address barriers to evidence-based care are warranted. Individuals who received MOUD differed from those who received no past-year drug treatment not only in terms of clinical need but also in terms of predisposing and enabling characteristics, highlighting a need for interventions and policies to increase MOUD uptake. Because most people encountered the health care and criminal legal systems, results suggest a need for cross-system integrated interventions to increase MOUD uptake.

## Notes

Supplement.
eMethods.
**eTable 1.** Sociodemographic Characteristics of Adults and Adolescents by Past-Year Treatment Utilization, 2019 **eTable 2.** Sociodemographic Characteristics of Adults Ages 18 and Older With Past-Year OUD Treatment Need, 2019 **eTable 3.** Likelihood of MOUD Treatment Among Adults With Past-Year OUD Treatment Need, Including Criminal Legal System Exposure

## References

[1] Mattson CL, Tanz LJ, Quinn K, Kariisa M, Patel P, Davis NL. Trends and geographic patterns in drug and synthetic opioid overdose deaths—United States, 2013-2019. MMWR Morb Mortal Wkly Rep. 2021;70(6):202-207. doi:10.15585/mmwr.mm7006a4 33571180 PMC7877587

[2] Gomes T, Tadrous M, Mamdani MM, Paterson JM, Juurlink DN. The burden of opioid-related mortality in the United States. JAMA Netw Open. 2018;1(2):e180217-e180217. doi:10.1001/jamanetworkopen.2018.0217 30646062 PMC6324425

[3] Walley AY, Xuan Z, Hackman HH, . Opioid overdose rates and implementation of overdose education and nasal naloxone distribution in Massachusetts: interrupted time series analysis. BMJ. 2013;346:f174. doi:10.1136/bmj.f174 23372174 PMC4688551

[4] Larochelle MR, Bernson D, Land T, . Medication for opioid use disorder after nonfatal opioid overdose and association with mortality: a cohort study. Ann Intern Med. 2018;169(3):137-145. doi:10.7326/M17-3107 29913516 PMC6387681

[5] Mattick RP, Breen C, Kimber J, Davoli M. Buprenorphine maintenance versus placebo or methadone maintenance for opioid dependence. *Cochrane Database of Syst Rev*. 2014(2):CD002207. doi:10.1002/14651858.CD002207.pub4 PMC10617756 24500948

[6] Volkow ND, Frieden TR, Hyde PS, Cha SS. Medication-assisted therapies–tackling the opioid-overdose epidemic. N Engl J Med. 2014;370(22):2063-2066. doi:10.1056/NEJMp1402780 24758595

[7] Krawczyk N, Mojtabai R, Stuart EA, . Opioid agonist treatment and fatal overdose risk in a state-wide US population receiving opioid use disorder services. Addiction. 2020;115(9):1683-1694. doi:10.1111/add.14991 32096302 PMC7426244

[8] Samples H, Williams AR, Crystal S, Olfson M. Impact of long-term buprenorphine treatment on adverse health care outcomes In Medicaid. Health Aff (Millwood). 2020;39(5):747-755. doi:10.1377/hlthaff.2019.01085 32364847 PMC7531057

[9] Wakeman SE, Larochelle MR, Ameli O, . Comparative effectiveness of different treatment pathways for opioid use disorder. JAMA Netw Open. 2020;3(2):e1920622-e1920622. doi:10.1001/jamanetworkopen.2019.20622 32022884 PMC11143463

[10] Askari MS, Martins SS, Mauro PM. Medication for opioid use disorder treatment and specialty outpatient substance use treatment outcomes: differences in retention and completion among opioid-related discharges in 2016. J Subst Abuse Treat. 2020;114:108028. doi:10.1016/j.jsat.2020.108028 32527510 PMC7328306

[11] Timko C, Schultz NR, Cucciare MA, Vittorio L, Garrison-Diehn C. Retention in medication-assisted treatment for opiate dependence: a systematic review. J Addict Dis. 2016;35(1):22-35. doi:10.1080/10550887.2016.1100960 26467975 PMC6542472

[12] Hadland SE, Bagley SM, Rodean J, . Receipt of timely addiction treatment and association of early medication treatment with retention in care among youths with opioid use disorder. JAMA Pediatr. 2018;172(11):1029-1037. doi:10.1001/jamapediatrics.2018.2143 30208470 PMC6218311

[13] Huhn AS, Hobelmann JG, Strickland JC, . Differences in availability and use of medications for opioid use disorder in residential treatment settings in the United States. JAMA Netw Open. 2020;3(2):e1920843-e1920843. doi:10.1001/jamanetworkopen.2019.20843 32031650 PMC8188643

[14] McGinty EE, Stone EM, Kennedy-Hendricks A, Bachhuber MA, Barry CL. Medication for opioid use disorder: a national survey of primary care physicians. Ann Intern Med. 2020;173(2):160-162. doi:10.7326/M19-3975 32311740 PMC8171002

[15] Mojtabai R, Mauro C, Wall MM, Barry CL, Olfson M. Medication treatment for opioid use disorders in substance use treatment facilities. Health Aff (Millwood). 2019;38(1):14-23. doi:10.1377/hlthaff.2018.05162 30615514 PMC6816341

[16] Barry CL, McGinty EE, Pescosolido BA, Goldman HH. Stigma, discrimination, treatment effectiveness, and policy: public views about drug addiction and mental illness. Psychiatr Serv. 2014;65(10):1269-1272. doi:10.1176/appi.ps.201400140 25270497 PMC4285770

[17] Kennedy-Hendricks A, Barry CL, Gollust SE, Ensminger ME, Chisolm MS, McGinty EE. Social stigma toward persons with prescription opioid use disorder: associations with public support for punitive and public health-oriented policies. Psychiatr Serv. 2017;68(5):462-469. doi:10.1176/appi.ps.201600056 28045350

[18] Stone EM, Kennedy-Hendricks A, Barry CL, Bachhuber MA, McGinty EE. The role of stigma in U.S. primary care physicians’ treatment of opioid use disorder. Drug Alcohol Depend. 2021;221:108627. doi:10.1016/j.drugalcdep.2021.108627 33621805 PMC8026666

[19] Mantha S, Mauro PM, Mauro CM, Martins SS. State criminal justice policy context and opioid agonist treatment delivery among opioid treatment admissions, 2015. Drug Alcohol Depend. 2020;206:107654. doi:10.1016/j.drugalcdep.2019.107654 31735533 PMC7377924

[20] Feder KA, Krawczyk N, Saloner B. Medication-assisted treatment for adolescents in specialty treatment for opioid use disorder. J Adolesc Health. 2017;60(6):747-750. doi:10.1016/j.jadohealth.2016.12.023 28258807 PMC6003902

[21] Olfson M, Zhang VS, Schoenbaum M, King M. Trends in buprenorphine treatment in the United States, 2009-2018. JAMA. 2020;323(3):276-277. doi:10.1001/jama.2019.18913 31961408 PMC6990679

[22] Krans EE, Kim JY, James AE III, Kelley D, Jarlenski MP. Medication-assisted treatment use among pregnant women with opioid use disorder. Obstet Gynecol. 2019;133(5):943-951. doi:10.1097/AOG.0000000000003231 30969219 PMC6483844

[23] Donohue JM, Jarlenski MP, Kim JY, ; Medicaid Outcomes Distributed Research Network (MODRN). Use of medications for treatment of opioid use disorder among US medicaid enrollees in 11 states, 2014-2018. JAMA. 2021;326(2):154-164. doi:10.1001/jama.2021.7374 34255008 PMC8278273

[24] Gordon AJ, Lo-Ciganic WH, Cochran G, . Patterns and quality of buprenorphine opioid agonist treatment in a large medicaid program. J Addict Med. 2015;9(6):470-477. doi:10.1097/ADM.0000000000000164 26517324

[25] Morgan JR, Schackman BR, Leff JA, Linas BP, Walley AY. Injectable naltrexone, oral naltrexone, and buprenorphine utilization and discontinuation among individuals treated for opioid use disorder in a United States commercially insured population. J Subst Abuse Treat. 2018;85:90-96. doi:10.1016/j.jsat.2017.07.001 28733097 PMC5750108

[26] Haffajee RL, Lin LA, Bohnert ASB, Goldstick JE. Characteristics of US counties with high opioid overdose mortality and low capacity to deliver medications for opioid use disorder. JAMA Netw Open. 2019;2(6):e196373-e196373. doi:10.1001/jamanetworkopen.2019.6373 31251376 PMC6604101

[27] Matusow H, Dickman SL, Rich JD, . Medication assisted treatment in US drug courts: results from a nationwide survey of availability, barriers and attitudes. J Subst Abuse Treat. 2013;44(5):473-480. doi:10.1016/j.jsat.2012.10.004 23217610 PMC3602216

[28] Bronson J, Stroop J, Zimmer S, Berzofsky M. Drug Use, Dependence, and Abuse Among State Prisoners and Jail Inmates, 2007-2009. Bureau of Justice Statistics, Office of Justice Programs, U.S. Department of Justice; 2017, NCJ 250546.

[29] Wu LT, Zhu H, Swartz MS. Treatment utilization among persons with opioid use disorder in the United States. Drug Alcohol Depend. 2016;169:117-127. doi:10.1016/j.drugalcdep.2016.10.015 27810654 PMC5223737

[30] Saloner B, Karthikeyan S. Changes in substance abuse treatment use among individuals with opioid use disorders in the United States, 2004-2013. JAMA. 2015;314(14):1515-1517. doi:10.1001/jama.2015.10345 26462001

[31] Substance Abuse and Mental Health Services Administration. Key Substance Use And Mental Health Indicators in the United States: Results from the 2019 National Survey on Drug Use and Health. Center for Behavioral Health Statistics and Quality, Substance Abuse and Mental Health Services Administration; 2020.

[32] Mark TL, Dilonardo J, Vandivort R, Miller K. Psychiatric and medical comorbidities, associated pain, and health care utilization of patients prescribed buprenorphine. J Subst Abuse Treat. 2013;44(5):481-487. doi:10.1016/j.jsat.2012.11.004 23265445

[33] Center for Behavioral Health Statistics and Quality. 2019 National Survey on Drug Use and Health: Methodological Summary and Definitions. Substance Abuse and Mental Health Services Administration; 2020.

[34] Jordan BK, Karg RS, Batts KR, Epstein JF, Wiesen C. A clinical validation of the National Survey on Drug Use and Health assessment of substance use disorders. Addict Behav. 2008;33(6):782-798. doi:10.1016/j.addbeh.2007.12.007 18262368

[35] Substance Abuse and Mental Health Services Administration. *Reliability of Key Measures in the National Survey on Drug Use and Health.* Substance Abuse and Mental Health Services Administration; 2010. 30199182

[36] Center for Behavioral Health Statistics and Quality. *2019 National Survey on Drug Use and Health (NSDUH): Methodological Resource Book, Section 8, Data Collection Final Report.* Substance Abuse and Mental Health Services Administration; 2020.

[37] von Elm E, Altman DG, Egger M, Pocock SJ, Gøtzsche PC, Vandenbroucke JP; STROBE Initiative. The Strengthening the Reporting of Observational Studies in Epidemiology (STROBE) statement: guidelines for reporting observational studies. Epidemiology. 2007;18(6):800-804. doi:10.1097/EDE.0b013e3181577654 18049194

[38] American Psychiatric Association. Diagnostic and Statistical Manual of Mental Disorders. 4th ed. American Psychiatric Publishing, Inc; 1994.

[39] Andersen RM. Revisiting the behavioral model and access to medical care: does it matter? J Health Soc Behav. 1995;36(1):1-10. doi:10.2307/2137284 7738325

[40] Krawczyk N, Williams AR, Saloner B, Cerdá M. Who stays in medication treatment for opioid use disorder? a national study of outpatient specialty treatment settings. J Subst Abuse Treat. 2021;126:108329. doi:10.1016/j.jsat.2021.108329 34116820 PMC8197774

[41] Hadland SE, Wharam JF, Schuster MA, Zhang F, Samet JH, Larochelle MR. Trends in receipt of buprenorphine and naltrexone for opioid use disorder among adolescents and young adults, 2001-2014. JAMA Pediatr. 2017;171(8):747-755. doi:10.1001/jamapediatrics.2017.0745 28628701 PMC5649381

[42] Squeglia LM, Fadus MC, McClure EA, Tomko RL, Gray KM. Pharmacological treatment of youth substance use disorders. J Child Adolesc Psychopharmacol. 2019;29(7):559-572. doi:10.1089/cap.2019.0009 31009234 PMC6727439

[43] Alinsky RH, Hadland SE, Matson PA, Cerda M, Saloner B. Adolescent-serving addiction treatment facilities in the United States and the availability of medications for opioid use disorder. J Adolesc Health. 2020;67(4):542-549. doi:10.1016/j.jadohealth.2020.03.005 32336560 PMC7508760

[44] Borodovsky JT, Levy S, Fishman M, Marsch LA. Buprenorphine treatment for adolescents and young adults with opioid use disorders: a narrative review. J Addict Med. 2018;12(3):170-183. doi:10.1097/ADM.0000000000000388 29432333 PMC5970018

[45] Camenga DR, Colon-Rivera HA, Muvvala SB. Medications for maintenance treatment of opioid use disorder in adolescents: a narrative review and assessment of clinical benefits and potential risks. J Stud Alcohol Drugs. 2019;80(4):393-402. doi:10.15288/jsad.2019.80.393 31495374

[46] Mauro PM, Samples H, Klein KS, Martins SS. Discussing drug use with health care providers is associated with perceived need and receipt of drug treatment among adults in the United States: we need to talk. Med Care. 2020;58(7):617-624. doi:10.1097/MLR.0000000000001340 32520836 PMC8112806

[47] Mauro PM, Askari MS, Han BH. Gender differences in any alcohol screening and discussions with providers among older adults in the United States, 2015 to 2019. Alcohol Clin Exp Res. 2021;45(9):1812-1820. doi:10.1111/acer.14668 34324221 PMC8908015

[48] Furr-Holden D, Milam AJ, Wang L, Sadler R. African Americans now outpace whites in opioid-involved overdose deaths: a comparison of temporal trends from 1999 to 2018. Addiction. 2021;116(3):677-683. doi:10.1111/add.15233 32852864

[49] Stein BD, Dick AW, Sorbero M, . A population-based examination of trends and disparities in medication treatment for opioid use disorders among Medicaid enrollees. Subst Abus. 2018;39(4):419-425. doi:10.1080/08897077.2018.1449166 29932847 PMC6309581

[50] Andraka-Christou B. Addressing racial and ethnic disparities in the use of medications for opioid use disorder. Health Aff (Millwood). 2021;40(6):920-927. doi:10.1377/hlthaff.2020.02261 34097509

[51] Substance Abuse and Mental Health Services Administration. Medicaid Coverage of Medication-Assisted Treatment for Alcohol and Opioid Use Disorders and of Medication for the Reversal of Opioid Overdose. Substance Abuse and Mental Health Services Administration; 2018.

[52] Costello AM. Mandatory Medicaid State Plan Coverage of Medication-Assisted Treatment. Centers for Medicare & Medicaid. December 30, 2020. Accessed October 28, 2021. https://www.medicaid.gov/federal-policy-guidance/downloads/sho20005.pdf

[53] Mark TL, Parish WJ, Zarkin GA. Association of formulary prior authorization policies with buprenorphine-naloxone prescriptions and hospital and emergency department use among Medicare beneficiaries. JAMA Netw Open. 2020;3(4):e203132. doi:10.1001/jamanetworkopen.2020.3132 32310285 PMC7171554

[54] Andrilla CHA, Moore TE, Patterson DG, Larson EH. Geographic distribution of providers with a DEA waiver to prescribe buprenorphine for the treatment of opioid use disorder: a 5-year update. J Rural Health. 2019;35(1):108-112. doi:10.1111/jrh.12307 29923637

[55] Saloner B, Lin L, Simon K. Geographic location of buprenorphine-waivered physicians and integration with health systems. J Subst Abuse Treat. 2020;115:108034. doi:10.1016/j.jsat.2020.108034 32600622 PMC7327133

[56] Andrilla CHA, Patterson DG. Tracking the geographic distribution and growth of clinicians with a DEA waiver to prescribe buprenorphine to treat opioid use disorder. J Rural Health. 2022;38(1):87-92. doi:10.1111/jrh.12569 33733547

[57] Abraham AJ, Andrews CM, Harris SJ, Friedmann PD. Availability of medications for the treatment of alcohol and opioid use disorder in the USA. Neurotherapeutics. 2020;17(1):55-69. doi:10.1007/s13311-019-00814-4 31907876 PMC7007488

[58] Thomas CP, Doyle E, Kreiner PW, . Prescribing patterns of buprenorphine waivered physicians. Drug Alcohol Depend. 2017;181:213-218. doi:10.1016/j.drugalcdep.2017.10.002 29096292

[59] Jones CM, McCance-Katz EF. Characteristics and prescribing practices of clinicians recently waivered to prescribe buprenorphine for the treatment of opioid use disorder. Addiction. 2019;114(3):471-482. doi:10.1111/add.14436 30194876

[60] Stein BD, Saloner B, Schuler MS, Gurvey J, Sorbero M, Gordon AJ. Concentration of patient care among buprenorphine-prescribing clinicians in the US. JAMA. 2021;325(21):2206-2208. doi:10.1001/jama.2021.4469 34061152 PMC8170540

[61] Volkow ND, Jones EB, Einstein EB, Wargo EM. Prevention and treatment of opioid misuse and addiction: a review. JAMA Psychiatry. 2019;76(2):208-216. doi:10.1001/jamapsychiatry.2018.3126 30516809

[62] D’Onofrio G, Edelman EJ, Hawk KF, . Implementation facilitation to promote emergency department-initiated buprenorphine for opioid use disorder: protocol for a hybrid type III effectiveness-implementation study (Project ED HEALTH). Implement Sci. 2019;14(1):48. doi:10.1186/s13012-019-0891-5 31064390 PMC6505286

[63] Kim HS, Samuels EA. Overcoming barriers to prescribing buprenorphine in the emergency department. JAMA Netw Open. 2020;3(5):e204996-e204996. doi:10.1001/jamanetworkopen.2020.4996 32391889

[64] Kilaru AS, Lubitz SF, Davis J, . A state financial incentive policy to improve emergency department treatment for opioid use disorder: a qualitative study. Psychiatr Serv. 2021;72(9):1048-1056. doi:10.1176/appi.ps.202000501 33593105 PMC12401733

[65] Cook BL, Alegría M. Racial-ethnic disparities in substance abuse treatment: the role of criminal history and socioeconomic status. Psychiatr Serv. 2011;62(11):1273-1281. doi:10.1176/ps.62.11.pss6211_1273 22211205 PMC3665009

[66] Krawczyk N, Picher CE, Feder KA, Saloner B. Only one in twenty justice-referred adults in specialty treatment for opioid use receive methadone or buprenorphine. Health Aff (Millwood). 2017;36(12):2046-2053. doi:10.1377/hlthaff.2017.0890 29200340 PMC6035729

[67] Midgette G, Caulkins JP, Reuter P. Pathways to drug prevalence estimation: synthesizing three comments on triangulation. Addiction. 2021;116(10):2615-2616. doi:10.1111/add.15607 34184339

[68] Reuter P, Caulkins JP, Midgette G. Heroin use cannot be measured adequately with a general population survey. Addiction. 2021;116(10):2600-2609. doi:10.1111/add.15458 33651441

[69] Radhakrishnan K. Significance of integration and use of multiple data sources for understanding substance use and mental health disorders. Addiction. 2021;116(10):2611-2613. doi:10.1111/add.15562 34036659

[70] Nesoff ED, Martins SS, Palamar JJ. Caution is necessary when estimating treatment need for opioid use disorder using national surveys. Am J Public Health. 2022;112(2):199-201. doi:10.2105/AJPH.2021.306624 35080936 PMC8802592

