# Errors in a Study Measure Definition

**DOI:** 10.1001/jamanetworkopen.2022.27817

**Published:** 2022-07-29

The Original Investigation titled “Use of Medication for Opioid Use Disorder Among US Adolescents and Adults With Need for Opioid Treatment, 2019,”^1^ published March 23, 2022, has been corrected to fix the definition of a study measure. Wherever *past-year *criminal legal system contact was indicated, it now reflects *lifetime* criminal legal system contact. Changes were made in the Abstract, Key Points, Methods section, Results section, and Figure title. This article has been corrected.^1^
