# Supplementary material for: Use of Medication for Opioid Use Disorder Among US Adolescents and Adults With Need for Opioid Treatment, 2019
Source: JAMA Netw Open. 2022 Mar 23;5(3):e223821. doi: 10.1001/jamanetworkopen.2022.3821 (PMC8943638; doi:10.1001/jamanetworkopen.2022.3821)
Supplement: Supplement. — eMethods. eTable 1. Sociodemographic Characteristics of Adults and Adolescents by Past-Year Treatment Utilization, 2019 eTable 2. Sociodemographic Characteristics of Adults Ages 18 and Older With Past-Year OUD Treatment Need, 2019 eTable 3. Likelihood of MOUD Treatment Among Adults With Past-Year OUD Treatment Need, Including Criminal Legal System Exposure [file jamanetwopen-e223821-s001.pdf]

Supplementary Online Content

Mauro PM, Gutkind S, Annunziato EM, Samples H. Use of medication for opioid use disorder among US adolescents and adults with need for opioid treatment, 2019. *JAMA Netw Open*. 2022;5(3):e223821. doi:10.1001/jamanetworkopen.2022.3821

eMethods.

eTable 1. Sociodemographic Characteristics of Adults and Adolescents by Past-Year Treatment Utilization, 2019

eTable 2. Sociodemographic Characteristics of Adults Ages 18 and Older With Past-Year OUD Treatment Need, 2019

eTable 3. Likelihood of MOUD Treatment Among Adults With Past-Year OUD Treatment Need, Including Criminal Legal System Exposure

This supplementary material has been provided by the authors to give readers additional information about their work.

eMethods

Inclusion criteria
Inclusion criteria for past-year OUD treatment need were as follows: (a) past-year OUD (i.e., past-year DSM-IV heroin abuse, heroin dependence, or imputation-revised prescription pain reliever abuse/dependence; (b) past-year MOUD (i.e., "medication to help reduce or stop your use of [heroin or prescription pain relievers]"), or (c) past-year last/current treatment episode for heroin and/or prescription pain relievers in a specialty setting.

(a) Past-year OUD included respondents who used heroin/non-medical prescription pain relievers in the past year and met at least one of the following DSM-IV criteria in the past 12-months (collected by substance):

- Heroin/prescription pain reliever abuse (eMethods Measure 1),
- Heroin/prescription pain reliever dependence (eMethods Measure 2).

(b) Past-year MOUD was based on 1 measure (eMethods Measure 3) and was asked of respondents who reported lifetime heroin/non-medical prescription pain reliever use and reported treatment for drug use in the past 12 months.

(c) Past-year last/current treatment episode for heroin and/or prescription pain relievers in a specialty setting was based on measures related to the last/current treatment for drug use among people who used heroin/prescription pain relievers in their lifetime (eMethods Measure 4)

- Lifetime use of heroin or non-medical prescription pain relievers,
  AND
- Last/current treatment or counseling for drug use was in the last year,
  AND
- Last/current treatment episode was to treat heroin/non-medical prescription pain reliever use, AND in a specialty setting.

Outcome
We differentiated MOUD, non-MOUD services, or no treatment for drug use (based on 2 measures) among people who needed OUD treatment in the past year (eMethods Measure 5)

- Past-year MOUD for heroin/prescription pain relievers
- Past-year treatment or counseling for drug use, regardless of alcohol use treatment status, not counting tobacco smoking cessation

Imputation-revised and recoded measures in the NSDUH
Variables that were imputation-revised according did not have any missingness. Substance dependence and abuse measures imputed in the 2019 NSDUH (CBHSQ, 2020) include: prescription pain relievers, prescription tranquilizers, prescription stimulants, prescription sedatives, hallucinogens, inhalants, and methamphetamine. These imputed measures had no missing values. Other variables without missingness include age, gender, race/ethnicity, education, insurance, income, and county.

Substance dependence and abuse measures that were recoded (but not imputed) include: heroin, alcohol, cannabis, and cocaine. For these, "no" responses and unknown values were combined. Additional information is provided in "Appendix D: Recoded substance dependence and abuse variable documentation" of the 2019 NSDUH Codebook (Center for Behavioral Health Statistics and Quality (CBHSQ), 2020). The past-year adult depressive episode variable was created using a mental illness predictor described in the "Recoded Adult Depression" module of the 2019 NSDUH Codebook (CBHSQ, 2020). Past-year last/current treatment variables, health contacts and criminal legal contacts were not imputed. For these, "no" responses and unknown values were combined.

Missing data in recoded variables that were not imputed in the NSDUH

Recoded variables used in our inclusion criteria may have affected who was included in our sample, because "no" responses and unknown values were combined. Of the N=56,136 in the 2019 NSDUH, n=55,649 were not included in our sample based on our inclusion criteria. Of all that did not meet our inclusion criteria, n=1031 (1.9%) had unknown values of past-year heroin use disorder or past-year last/current opioid specialty treatment and did not otherwise meet inclusion criteria, as described below.

- Past-year Prescription pain reliever use abuse/dependence
    - n=0 missing, imputation revised data
- Past-year Heroin abuse/dependence
    - n=40 (0.1% of all who did not meet inclusion criteria) had unknown/missing heroin use disorder measures and were excluded because they did not meet any inclusion criteria.
- Past-year MOUD
    - n=0 missing, recoded data, source information not available in the public file
    - "No/unknown" responses combined in public data
- Past-year Last/current specialty treatment for opioids
    - n=991 (1.8% of all who did not meet inclusion criteria) had unknown/missing measures only for past-year last/current specialty treatment for opioids after accounting for observations also missing heroin use disorder. Of these, 907 were missing information on whether the last/current treatment was for opioids
    - Only n=68 of these 991 (0.1% of all who did not meet inclusion criteria) reported lifetime heroin/non-medical prescription opioid use and were excluded because they did not meet any inclusion criteria.

We examined opioid use (based on imputation-revised recency data for heroin and non-medical prescription pain relievers) among observations excluded due to missing data in our inclusion criteria. *Of the 1031 observations with missing inclusion criteria data, 952 (92.3%) did not indicate lifetime heroin/non-medical prescription pain reliever use and therefore would likely not need OUD treatment.*

- 11 observations (1.1% of missing, 0.02% of all who did not meet inclusion criteria) unknown/missing heroin use disorder data reported lifetime heroin use (who did not meet prescription OUD criteria), including 1 reporting past-year heroin (0.1% of missing)
- 68 observations (6.6% of missing, 0.1% of all who did not meet inclusion criteria) unknown/missing past-year last/current specialty treatment episode for opioids (and were not missing OUD criteria) reported lifetime heroin/NMPO use, including 38 reporting past-year heroin/NMPO but no OUD (3.7% of missing, 0.1% of all who did not meet inclusion criteria)

For the full computer-assisted interview document and codebook, please see:

Center for Behavioral Health Statistics and Quality. (2018). 2019 National Survey on Drug Use and Health (NSDUH): CAI Specifications for Programming (English Version). Substance Abuse and Mental Health Services Administration, Rockville, MD. https://www.samhsa.gov/data/sites/default/files/reports/rpt23057/NSDUHmrbCAISpecs2019.pdf

Center for Behavioral Health Statistics and Quality. (2020). 2019 National Survey on Drug Use and Health Public Use File Codebook, Substance Abuse and Mental Health Services Administration, Rockville, MD

eMethods Measure 1. DSM-IV heroin abuse or prescription pain reliever abuse

To be classified with heroin abuse or prescription pain reliever abuse, a respondent must have met **one or more** of the four criteria listed below in the past 12-months. Separate sets of otherwise identical questions were asked for heroin abuse and prescription pain reliever abuse among people who reported past 12-month use of heroin or non-medical prescription pain reliever use, respectively.
Note: Non-medical prescription pain reliever use included people who reported using prescription pain relievers "in any way a doctor did not direct you to use it" in the past 12-months. This included 36 different types of prescription pain relievers (e.g., Vicodin, Lortab). The NSDUH imputation revised variable was available for prescription pain reliever abuse only and had no missing/unknown values. Heroin abuse was not imputed, so "no" responses and unknown values were combined in the NSDUH.

DSM-IV Heroin/Prescription Pain Reliever Abuse Criteria (at least 1 in the past year)	Question(s)	Answer(s) to meet criterion
Serious problems at home, work, or school caused by using **[heroin/prescription pain relievers],** such as<br>− neglecting their children<br>− missing work or school<br>− doing a poor job at work or school<br>− losing a job or dropping out of school	"Sometimes people who use **[heroin/prescription pain relievers]** have serious problems at home, work or school - such as:<br>- neglecting their children<br>- missing work or school<br>- doing a poor job at work or school<br>- losing a job or dropping out of school<br>During the past 12 months, did using **[heroin/prescription pain relievers]** cause you to have serious problems like this either at home, work, or school?"	"Yes"
Used **[heroin/prescription pain relievers]** regularly and then did something that might have put you in physical danger	"During the past 12 months, did you regularly use **[heroin/prescription pain relievers]** and then do something where using **[heroin/prescription pain relievers]** might have put you in physical danger?"	"Yes"
Use of **[heroin/prescription pain relievers]** caused you to do things that repeatedly got you in trouble with the law	"During the past 12 months, did using **[heroin/prescription pain relievers]** cause you to do things that repeatedly got you in trouble with the law?"	"Yes"
Continued to use **[heroin/prescription pain relievers]** even though you thought using **[heroin/prescription pain relievers]** caused problems	"During the past 12 months, did you have any problems with family or friends that were probably caused by your use of **[heroin/prescription pain relievers]**?"<br><br>"Did you continue to use **[heroin/prescription pain relievers]** even though you thought this caused problems with family or friends?"	"Yes" to both

eMethods Measure 2. DSM-IV Heroin dependence or prescription pain reliever dependence

To be classified with heroin dependence or prescription pain reliever dependence, a respondent must have met **three or more** of the dependence criteria listed below in the past 12-months. Separate sets of otherwise identical questions were asked for heroin dependence and prescription pain reliever dependence among people who reported past 12-month use of heroin or non-medical prescription pain reliever use, respectively.
Note: Non-medical prescription pain reliever use included people who reported using prescription pain relievers "in any way a doctor did not direct you to use it" in the past 12 months. This included 36 different types of prescription pain relievers (e.g., Vicodin, Lortab). The NSDUH imputation revised variable was available for prescription pain reliever dependence only. Heroin dependence was not imputed, so "no" responses and unknown values were combined in the NSDUH.

DSM-IV Heroin/Prescription Pain Reliever Dependence Criteria (at least 3 in the past year)	Question(s)	Answer(s) to meet criterion
Spent a great deal of time over a period of a month or more getting, using, or getting over the effects of **[heroin/prescription pain relievers]**	"During the past 12 months, was there a month or more when you spent a lot of time getting or using **[heroin/prescription pain relievers]** ?"<br><br>"During the past 12 months, was there a month or more when you spent a lot of your time getting over the effects of the **[heroin/prescription pain relievers]** you used?	"Yes" to at least one
Unable to keep set limits on d **[heroin/prescription pain reliever]** use or used more often than intended	"Were you able to keep to the limits you set, or did you often use **[heroin/prescription pain relievers]** more than you intended to?"	"Often used more than intended"
Needed to use **[heroin/prescription pain relievers]** more than before to get desired effects or noticed that same amount of **[heroin/prescription pain reliever]** use had less effect than before	"During the past 12 months, did you need to use more **[heroin/prescription pain relievers]** than you used to in order to get the effect you wanted?"<br><br>"During the past 12 months, did you notice that using the same amount of **[heroin/prescription pain relievers]** had less effect on you than it used to?"	"Yes" to at least one
Unable to cut down or stop using **[heroin/prescription pain relievers]** every time tried or wanted to	"During the past 12 months, were you able to cut down or stop using **[heroin/prescription pain relievers]** every time you wanted to or tried to?"	"No"
Continued to use **[heroin/prescription pain relievers]** even though use was causing problems with emotions, nerves, mental health, or physical problems	"Did you continue to use **[heroin/prescription pain relievers]** even though you thought this was causing you to have problems with your emotions, nerves, or mental health?"<br><br>"Did you continue to use **[heroin/prescription pain relievers]** even though you thought this was causing you to have physical problems?"	"Yes" to at least one
Reduced or gave up participation in important activities due to **[heroin/prescription pain reliever]** use	"This question is about important activities such as working, going to school, taking care of children, doing fun things such as hobbies and sports, and spending time with friends and family. During the past 12 months, did using **[heroin/prescription pain relievers]** cause you to give up or spend less time doing these types of important activities?"	"Yes"

DSM-IV Heroin/Prescription Pain Reliever Dependence Criteria (at least 3 in the past year)	Question(s)	Answer(s) to meet criterion
Experienced three or more **[heroin/prescription pain relievers]** withdrawal symptoms at the same time that lasted longer than a day after **[heroin/prescription pain reliever]** use was cut back or stopped.	"Please look at the symptoms listed below. During the past 12 months, did you have 3 or more of these symptoms at the same time that lasted for longer than a day after you cut back or stopped using **[heroin/prescription pain relievers]**?<br><br>- Feeling kind of blue or down<br>- Vomiting or feeling nauseous<br>- Having cramps or muscle aches<br>- Having teary eyes or a runny nose<br>- Feeling sweaty, having enlarged eye pupils, or having body hair standing up on your skin - Having diarrhea<br>- Yawning<br>- Having a fever<br>- Having trouble sleeping"	"Yes"

eMethods Measure 3. Medication for opioid use disorder (MOUD)

The variable used to classify past-year MOUD use specifically asked respondents about "medication-assisted treatment" received for heroin or prescription pain reliever use, not for alcohol use. Medication for alcohol use disorder was assessed separately from medication assisted treatment for heroin or prescription pain relievers and was not included in our study.
Note: Receipt of MOUD for heroin or prescription pain reliever use was ascertained only for respondents who reported lifetime heroin or non-medical prescription pain reliever use *and* past-year treatment/counseling for drug use. MOUD was not imputed, so "no" responses and unknown values were combined in the NSDUH.

Criteria	Question(s)	Answer(s) to meet criterion
Lifetime heroin use or non-medical prescription pain reliever use	"Have you ever, even once, used heroin?"<br><br>"In the past 12 months, did you use (specific prescription pain reliever name) in any way a doctor did not direct you to use it?" Asked for 36 specific and other prescription pain relievers<br><br>"Have you ever, even once, used any prescription pain reliever in any way a doctor did not direct you to use it?"	"Yes" to at least one
Treatment for drug use in the past 12 months	"Have you ever received treatment or counseling for your use of alcohol or any drug, not counting cigarettes?"<br><br>"During the past 12 months, that is, since [DATEFILL], have you received treatment or counseling for your use of alcohol or any drug, not counting cigarettes?"	"Yes" to both
	"During the past 12 months when you received treatment, was the treatment for alcohol use only, drug use only, or both alcohol and drug use?"	"Drug use only" or "Both alcohol and drug use"
MOUD in the past 12 months<br><br>(does not include medication for alcohol use disorder)	"The next question is about medication- assisted treatment prescribed by a doctor or other health professional to help reduce or stop your use of **[heroin/prescription pain relievers].** It is different from medications given to stop a drug overdose. Some examples of medication-assisted treatment include:<br>• Buprenorphine or buprenorphine-naloxone pills or film taken by mouth, also known as Suboxone, Zubsolv, Bunavail, or Subutex<br>• Injectable buprenorphine, also known as Sublocade<br>• Buprenorphine implant placed under the skin, also known as Probuphine<br>• Methadone<br>• Naltrexone pills, also known as ReVia or Trexan<br>• Injectable naltrexone, also known as Vivitrol<br>In the past 12 months, did you use medication to help reduce or stop your use of **[heroin/prescription pain relievers]**?"	"Yes"

eMethods Measure 4. Last/current treatment episode for heroin and/or prescription pain relievers in a specialty setting.

Respondents were included in the sample with OUD treatment need if they reported that their most recent treatment episode for their drug use in the past year was received in a specialty setting, specifically to treat heroin and/or prescription pain reliever use. These variables were not imputed, so “no” responses and unknown values were combined.

Criteria	Question(s)	Answer(s) to meet criterion
Lifetime or past-year use of heroin or non-medical prescription pain reliever use	“Have you ever, even once, used heroin?”<br><br>“In the past 12 months, did you use [specific prescription pain reliever name] in a way a doctor did not direct you to use it?” for 36 specified and “other” prescription pain relievers<br><br>“Have you ever, even once, used any prescription pain reliever in any way a doctor did not direct you to use it?”	“Yes” to at least one
Current or most recent **treatment** or counseling for alcohol or drug use was **within the past 12 months.**	“Have you ever received treatment or counseling for your use of alcohol or any drug, not counting cigarettes?”	“Yes”
	“How long has it been since you were last in treatment or counseling for your alcohol or drug use, not counting cigarettes?”	One of the following:<br>-“Still in treatment”<br>-“Within the past 30 days”<br>-“More than 30 days ago but within the past 12 months”
Current or most recent **treatment** or counseling is/was for **prescription pain relievers or heroin**.	“The last time you entered treatment, did you receive treatment or counseling for your use of **[heroin/prescription pain relievers]**?”<br><br>“Are you currently receiving treatment or counseling for your use of **[heroin/prescription pain relievers]**?”	“Yes” to at least one
Current or most recent **treatment** or counseling is/was in a **specialty setting**.	“What was the main place where you received treatment the last time you started treatment/are currently receiving treatment for your alcohol or other drug use, not counting cigarettes?”	One of the following:<br>- “A hospital overnight as an inpatient”<br>- “A res drug/alcohol rehab facility overnight”<br>- “A drug or alcohol rehab facility as an outpatient”<br>- “A mental health center or facility as outpatient”<br>- “Rehab/intervention fac/prgm, ptnt status unspcfd”<br>-“Methadone clinic/program”

eMethods Measure 5. Categorical outcome of medication for opioid use disorder (MOUD), non-MOUD services, or no treatment.

The categorical outcome differentiated people reporting past-year MOUD from those reporting non-MOUD services and those reporting no treatment. This hierarchical coding prioritized MOUD above non-MOUD services for respondents with both types of treatment. See eMethods Measures 1-4 for additional information regarding the sequence of questions leading up to the questions used to derive the outcome.

**Categories**	Question(s)	Answer(s) per category
**MOUD**	"The next question is about medication- assisted treatment prescribed by a doctor or other health professional to help reduce or stop your use of **[heroin/prescription pain relievers]**. It is different from medications given to stop a drug overdose. Some examples of medication- assisted treatment include:<br>• Buprenorphine or buprenorphine-naloxone pills or film taken by mouth, also known as Suboxone, Zubsolv, Bunavail, or Subutex<br>• Injectable buprenorphine, also known as Sublocade<br>• Buprenorphine implant placed under the skin, also known as Probuphine<br>• Methadone<br>• Naltrexone pills, also known as ReVia or Trexan<br>• Injectable naltrexone, also known as Vivitrol<br>In the past 12 months, did you use medication to help reduce or stop your use of **[heroin/prescription pain relievers]**?"	"Yes"
**Non-MOUD services**<br><br>(if reported no past-year MOUD)	"During the past 12 months when you received treatment, was the treatment for alcohol use only, drug use only, or both alcohol and drug use?"	"Drug use only" or<br>"Both alcohol and drug use"
**No treatment**	All reporting no past-year MOUD and no past-year non-MOUD services	

eTable 1. Sociodemographic characteristics of adults and adolescents by past-year treatment utilization, 2019

	Overall sample with OUD treatment need	Past-year drug treatment utilization		
		No treatment	Non-MOUD services	MOUD
**Characteristics**	Unweighted N (Wt. Col %)	Unweighted N (Wt. Col %)	Unweighted N (Wt. Col %)	Unweighted N (Wt. Col %)
**Predisposing**				
Age in years				
12-17	49 (4.17)	41 (6.43)	8 (3.34)	0 (0.00)
18-25	121 (11.41)	72 (12.32)	23 (12.33)	26 (9.06)
26-34	136 (29.30)	51 (21.06)	24 (32.14)	61 (44.65)
35-49	136 (31.73)	62 (27.24)	23 (42.18)	51 (35.19)
50+	45 (23.38)	31 (32.95)	5 (10.01)	9 (11.10)
Gender				
Male	240 (55.52)	116 (52.43)	47 (68.83)	77 (54.56)
Female	247 (44.48)	141 (47.57)	36 (31.17)	70 (45.44)
Race/ethnicity				
Hispanic, any race	51 (8.01)	32 (10.08)	13 (7.08)	6 (4.26)
Non-Hispanic Black	51 (9.93)	33 (11.00)	9 (11.34)	9 (6.97)
Non-Hispanic white	344 (74.57)	165 (70.15)	53 (75.43)	126 (83.18)
Non-Hispanic other	41 (7.49)	27 (8.77)	8 (6.15)	6 (5.59)
Education (18+)				
High school or less	231 (44.24)	111 (39.99)	48 (63.53)	72 (42.16)
Some college or more	207 (55.76)	105 (60.01)	27 (36.47)	75 (57.84)
**Enabling**				
Insurance categories				
Private only	138 (26.13)	94 (31.34)	17 (18.20)	27 (19.80)
Any public	254 (53.74)	111 (46.57)	43 (54.54)	100 (68.02)
Uninsured/other	95 (20.12)	52 (22.09)	23 (27.26)	20 (12.18)
Income				
$0-$19,999	164 (33.40)	66 (25.54)	34 (41.30)	64 (45.17)
$20,000- $49,999	167 (30.57)	93 (32.40)	29 (29.70)	45 (27.27)
$50,000- $74,999	68 (14.14)	40 (16.63)	10 (15.54)	18 (8.25)
$75,000+	88 (21.90)	58 (25.42)	10 (13.47)	20 (19.31)
Urbanicity				
Large Metro	200 (52.25)	112 (54.93)	26 (37.75)	62 (54.72)
Small Metro	186 (34.20)	92 (31.38)	40 (49.22)	54 (31.73)
Non-Metro	101 (13.55)	53 (13.69)	17 (13.03)	31 (13.55)
**Need**				
Any OUD	369 (77.1)	257 (100.00)	41 (44.59)	71 (47.97)
Prescription OUD only	262 (56.81)	212 (83.47)	21 (22.96)	29 (20.72)
Heroin use disorder only	69 (12.12)	30 (12.56)	14 (12.76)	25 (10.86)
Co-occurring heroin and prescription OUD	38 (8.17)	15 (3.97)	6 (8.87)	17 (16.39)
Other co-occurring substance use disorder	386 (79.97)	243 (94.17)	59 (65.18)	84 (58.97)
Major depressive episode (18+)	171 (38.65)	82 (35.00)	28 (38.39)	61 (46.27)

Notes: Weighted N=2,206,169; Unweighted N=487; n<5 observations masked due to small cell size in the unweighted sample. MOUD=Medication for Opioid Use Disorder; OUD=opioid use disorder; Self-reported race/ethnicity included Hispanic/Latinx, non-Hispanic Black, non-Hispanic white, and non-Hispanic other

(including Asian, Native American or Alaska Native, Native Hawaiian, Pacific Islander, or multiracial). (18+)=characteristic restricted to adults ages 18 and older. Other Co-occurring Substance Use Disorder=includes one or more of the following past-year substance use disorders: alcohol, cannabis, cocaine, hallucinogens, inhalants, methamphetamine, tranquilizers, stimulants, sedatives, and psychedelics.

eTable 2. Sociodemographic characteristics of adults ages 18 and older with past-year OUD treatment need, 2019

Characteristics	Adult subsample with OUD treatment need	Past-year drug treatment utilization			
		No treatment	Non-MOUD services	MOUD	
	Unweighted N (Wt. Col. %)	Unweighted N (Wt. Row %)	Unweighted N (Wt. Row %)	Unweighted N (Wt. Row %)	*P* value
**Total (row %)**	438 (100.00)	216 (55.63)	75 (15.39)	147 (28.98)	
**Predisposing**					
Age in years					
18-25	**121 (11.91)**	**72 (61.47)**	**23 (16.48)**	**26 (22.05)**	.01
26-34	**136 (30.58)**	**51 (40.95)**	**24 (16.73)**	**61 (42.32)**	
35-49	**136 (33.11)**	**62 (48.91)**	**23 (20.28)**	**51 (30.80)**	
50+	**45 (24.40)**	**31 (80.28)**	**5 (6.53)**	**9 (13.19)**	
Gender					
Male	222 (56.05)	103 (52.79)	42 (19.00)	77 (28.22)	.13
Female	216 (43.95)	113 (59.25)	33 (10.79)	70 (29.96)	
Race/ethnicity					
Hispanic, any race	44 (7.69)	25 (69.28)	13 (14.65)	6 (16.07)	.70
Non-Hispanic Black	37 (9.22)	21 (59.69)	7 (18.40)	9 (21.91)	
Non-Hispanic white	318 (75.42)	145 (52.67)	47 (15.36)	126 (31.96)	
Non-Hispanic other	39 (7.67)	25 (66.11)	8 (12.76)	6 (21.13)	
Education					
High school or less	**231 (44.24)**	**111 (50.28)**	**48 (22.10)**	**72 (27.62)**	.03
Some college or more	**207 (55.76)**	**105 (59.87)**	**27 (10.07)**	**75 (30.06)**	
**Enabling**					
Insurance categories					
Private only	**119 (25.37)**	**76 (66.44)**	**16 (10.94)**	**27 (22.62)**	.02
Any public	**228 (54.24)**	**92 (48.40)**	**36 (15.25)**	**100 (36.35)**	
Uninsured/other	**91 (20.38)**	**48 (61.40)**	**23 (21.29)**	**20 (17.31)**	
Detailed insurance indicators*					
Medicaid	**208 (41.95)**	**78 (38.13)**	**34 (16.78)**	**96 (45.09)**	<.001
Medicare	**36 (14.93)**	**23 (78.81)**	**5 (10.68)**	**8 (10.51)**	<.001
Champus/Military	12 (5.59)	6 (71.98)	<5 (12.84)	5 (15.17)	.62
Private	144 (30.76)	86 (61.53)	21 (14.25)	37 (24.22)	.41
Other	9 (1.85)	<5 (62.41)	<5 (15.51)	<5 (22.07)	.90
No insurance	**82 (18.53)**	**44 (61.30)**	**21 (21.87)**	**17 (16.84)**	.05
Income					
$0-$19,999	154 (33.85)	59 (42.64)	31 (18.68)	64 (38.68)	.13
$20,000- $49,999	144 (30.02)	74 (58.82)	25 (14.85)	45 (26.33)	
$50,000- $74,999	63 (14.35)	35 (66.09)	10 (17.24)	18 (16.66)	
$75,000+	77 (21.79)	48 (64.52)	9 (9.80)	20 (25.69)	
Urbanicity					
Large Metro	178 (51.88)	92 (58.10)	24 (11.33)	62 (30.57)	.35
Small Metro	170 (34.53)	79 (51.46)	37 (21.90)	54 (26.64)	
Non-Metro	90 (13.60)	45 (56.77)	14 (14.34)	31 (28.89)	
**Need**					
Any OUD	**324 (76.23)**	**216 (72.97)**	**37 (8.79)**	**71 (18.24)**	<.001
Prescription OUD only	**217 (55.06)**	**171 (83.18)**	**17 (5.91)**	**29 (10.91)**	<.001

Heroin use disorder only	**69 (12.64)**	**30 (59.04)**	**14 (16.06)**	**25 (24.90)**	
Co-occurring heroin and prescription OUD	**38 (8.53)**	**15 (27.71)**	**6 (16.56)**	**17 (55.73)**	
Other co-occurring substance use disorder	**337 (79.10)**	**202 (65.94)**	**51 (12.45)**	**84 (21.61)**	<.001
Major depressive episode	171 (40.33)	82 (51.60)	28 (15.15)	61 (33.25)	.46

Notes: Weighted N=2,114,089; *detailed insurance indicators are not mutually exclusive; n<5 observations masked due to small cell size. MOUD=Medication for Opioid Use Disorder; OUD=opioid use disorder; Other Co-occurring Substance Use Disorder=includes one or more of the following past-year substance use disorders: alcohol, cannabis, cocaine, hallucinogens, inhalants, methamphetamine, tranquilizers, stimulants, sedatives, and psychedelics. Self-reported race/ethnicity included Hispanic/Latinx, non-Hispanic Black, non-Hispanic white, and non-Hispanic other (including Asian, Native American or Alaska Native, Native Hawaiian, Pacific Islander, or multiracial). Bold indicates design-based *P*<.05 with Rao-Scott adjustment.

eTable 3. Likelihood of MOUD treatment among adults with past-year OUD treatment need, including criminal legal system exposure

	**MOUD vs. No treatment**	**MOUD vs. Non-MOUD services**
**Characteristics**	aRRR (95% CI)	aRRR (95% CI)
**Predisposing**		
Age categories		
18-25	1.00 (Ref)	1.00 (Ref)
26-34	0.89 (0.26, 2.99)	0.88 (0.26, 3.00)
35-49	0.71 (0.23, 2.20)	0.63 (0.22, 1.86)
50+	**0.24 (0.07, 0.84)**	1.08 (0.40, 2.94)
Race/ethnicity		
Hispanic	0.51 (0.12, 2.12)	0.50 (0.12, 2.06)
Non-Hispanic Black	0.73 (0.24, 2.18)	0.45 (0.12, 1.73)
Non-Hispanic white	1.00 (Ref)	1.00 (Ref)
Non-Hispanic other	0.39 (0.13, 1.22)	0.38 (0.08, 1.71)
Gender		
Male	1.00 (Ref)	1.00 (Ref)
Female	**0.55 (0.31, 0.99)**	1.74 (0.76, 3.99)
Education		
High school or less	1.00 (Ref)	1.00 (Ref)
Some college or more	**2.20 (1.14, 4.24)**	**3.08 (1.36, 6.97)**
**Enabling**		
Insurance		
Medicaid	1.00 (Ref)	1.00 (Ref)
Medicare	0.29 (0.07, 1.13)	0.43 (0.12, 1.58)
Champus/Military	0.92 (0.17, 5.09)	0.57 (0.07, 4.47)
Private	**0.39 (0.18, 0.85)**	0.58 (0.18, 1.89)
Other	0.28 (0.06, 1.28)	0.28 (0.04, 1.99)
None	**0.20 (0.05, 0.80)**	**0.25 (0.07, 0.83)**
Income		
$0-$19,999	1.00 (Ref)	1.00 (Ref)
$20,000- $49,999	0.58 (0.28, 1.19)	0.87 (0.31, 2.43)
$50,000- $74,999	**0.24 (0.10, 0.60)**	0.48 (0.12, 1.93)
$75,000+	0.41 (0.13, 1.27)	1.06 (0.23, 4.84)
Urbanicity		
Large Metro	1.00 (Ref)	1.00 (Ref)
Small Metro	1.02 (0.53, 1.96)	0.46 (0.20, 1.06)
Non-Metro	0.76 (0.29, 2.00)	0.80 (0.25, 2.63)
**Need**		
Both heroin/prescription OUD	**4.59 (1.24, 16.95)**	2.22 (0.41, 12.09)
Co-occurring substance use disorder	**0.08 (0.03, 0.20)**	0.67 (0.20, 2.31)
Major depressive episode	1.66 (0.89, 3.11)	0.99 (0.46, 2.13)
**Criminal legal system exposure**	**3.73 (1.78, 7.80)**	1.89 (0.68, 5.26)

Notes: Weighted N=2,114,089; unweighted N=438; MOUD=Medication for Opioid Use Disorder; OUD=opioid use disorder; aRRR= adjusted relative risk ratio from the multinomial model with a categorical drug treatment/OUD outcome. Adolescents 12-17 were excluded from the model because of collinearity with the outcome (i.e., no MOUD). Self-reported race/ethnicity included Hispanic/Latinx, non-Hispanic Black, non-Hispanic white, and non-Hispanic other (including Asian, Native American or Alaska Native, Native Hawaiian, Pacific Islander, or multiracial). Bold indicates design-based *P*<.05
